# Costs and cost-effectiveness of LEEP versus cryotherapy for treating cervical dysplasia among HIV-positive women in Johannesburg, South Africa

**DOI:** 10.1371/journal.pone.0203921

**Published:** 2018-10-11

**Authors:** Naomi Lince-Deroche, Craig van Rensburg, Jaqueline Roseleur, Busola Sanusi, Jane Phiri, Pam Michelow, Jennifer S. Smith, Cindy Firnhaber

**Affiliations:** 1 Health Economics and Epidemiology Research Office, Department of Internal Medicine, School of Clinical Medicine, Faculty of Health Sciences, University of the Witwatersrand, Johannesburg, South Africa; 2 Gillings School of Public Health, University of North Carolina, Chapel Hill, NC, United States of America; 3 Cytology Unit, National Health Laboratory Service and Department of Anatomical Pathology, Faculty of Health Sciences, University of the Witwatersrand, Johannesburg, South Africa; 4 Lineberger Comprehensive Cancer Center, University of North Carolina, Chapel Hill, NC, United States of America; 5 Right to Care, Johannesburg, South Africa; 6 Clinical HIV Research Unit, Department of Internal Medicine, Faculty of Health Sciences, University of Witwatersrand, Johannesburg, South Africa; Katholieke Universiteit Leuven Rega Institute for Medical Research, BELGIUM

## Abstract

**Background:**

Cervical cancer incidence is significant in countries, such as South Africa, with high burdens of both HIV and human papillomavirus (HPV). Cervical cancer is largely preventable if dysplasia is diagnosed and treated early, but there is debate regarding the best approaches for screening and treatment, especially for low-resource settings. Currently South Africa provides Pap smears followed by colposcopic biopsy and LEEP if needed in its public health facilities. We estimated the costs and cost-effectiveness of two approaches for treating cervical intraepithelial neoplasia grade 2 or higher (CIN2+) among HIV-infected women, most of whom were taking antiretroviral treatment, at a public HIV treatment facility in Johannesburg, South Africa.

**Methods:**

Method effectiveness was derived from an intention-to-treat analysis of data gathered in a clinical trial completed previously at the study facility. In the trial, women who were diagnosed with CIN2+ and eligible for cryotherapy were randomized to cryotherapy or LEEP. If women were CIN2+ at six months as determined via Pap smear and colposcopic biopsy, all women—regardless of their original treatment assignment—received LEEP. “Cure” was then defined as the absence of disease at 12 months based on Pap smear and colposcopic biopsy. Health service costs were estimated using micro-costing between June 2013 and April 2014. Capital costs were annualized using a discount rate of 3%. Two different service volume scenarios were considered, and results from an as-treated analysis were considered in sensitivity analysis.

**Results:**

In total, 166 women with CIN2+ were enrolled (86 had LEEP; 80 had cryotherapy). At 12 months, cumulative loss to follow-up was 12.8% (11/86) for the LEEP group and 13.8% (11/80) for cryotherapy. Based on the unadjusted intention-to-treat analysis conducted for this economic evaluation, there was no significant difference in efficacy. At 12 months, 83.8% (95% CI 73.8–91.1) of women with CIN2+ at baseline and randomized to cryotherapy were free of CIN2+ disease. In contrast, 76.7% (95% CI 66.4–85.2) of women assigned to LEEP were free from disease. On average, women initially treated with cryotherapy were less costly per patient randomized at US$ 118.00 (113.91–122.10), and per case “cured” at US$ 140.90 (136.01–145.79). Women in the LEEP group cost US$ 162.56 (157.90–167.22) per patient randomized and US$ 205.59 (199.70–211.49) per case cured. In the as-treated analysis, which was based on trial data, LEEP was more efficacious than cryotherapy; however, the difference was not significant. Cryotherapy remained more cost-effective than LEEP in all sensitivity and scenario analyses.

**Conclusions:**

For this cost-effectiveness analysis, using an intention-to-treat approach and taking into consideration uncertainty in the clinical and cost outcomes, a strategy involving cryotherapy plus LEEP if needed at six months was dominant to LEEP plus LEEP again at six months if needed for retreatment. However, compared to other studies comparing LEEP and cryotherapy, the efficacy results were low in both treatment groups–possibly due to the HIV-positivity of the participants. Further research is needed, but at present choosing the “right” treatment option may be less important than ensuring access to treatment and providing careful monitoring of treatment outcomes.

## Introduction

Cervical cancer incidence is high in countries with high burdens of both HIV and human papillomavirus (HPV) [1]. Most cervical cancer is caused by persistent HPV infection [2,3], and HIV increases both susceptibility to HPV and the risk of HPV persistence [4,5].

HIV and HPV infection rates in South Africa are among the highest globally. Among women with normal cytology and unknown HIV status, Allan et al (2008) found an HPV prevalence of 20.4%, and among women with high-grade squamous intraepithelial lesions (HSIL) HPV prevalence was 83% [6]. Adult HIV prevalence nationally in South Africa is 19.2% [7]; however, among pregnant women it was 29.5% in 2012 [8]. HPV infection rates for HIV-positive women in South Africa have been documented at 61% [9].

Cervical cancer is the second most common cancer among women in South Africa, and results in the most cancer-related deaths [10]. This is despite a national policy that provides for free access to screening and treatment in the public sector [11,12], where roughly 80% of the population accesses health care [13]. The national policy provides for free Pap smears in the public sector at 10-yearly intervals between ages 30 and 50 in HIV-negative women. Also, if a woman tests positive for HIV, she can immediately have a Pap smear, and follow-up visits are done at three- or one-yearly intervals depending on the results [11,12,14]. For any woman, if the Pap smear reveals persistent low-grade dysplasia, high-grade dysplasia or possible cancer, South Africa’s national policy recommends a colposcopic biopsy to clarify the diagnosis. Then, the treatment recommended for high-grade pre-cancer, or cervical intraepithelial neoplasia grade two or three (CIN2+), is a loop electrosurgical excision procedure (LEEP). LEEP utilizes a wire loop charged with electricity to excise the transformation zone of the cervix. The procedure requires trained doctors and local anesthesia [15].

In many resource-poor settings globally, an alternative approach is used for screening and treatment–visual inspection with acetic acid (VIA) followed by cryotherapy [16]. VIA and cryotherapy are available in very limited locations in South Africa–almost exclusively where the services are supported by NGOs. Cryotherapy involves the freezing of a lesion with a probe using nitrogen dioxide or carbon dioxide. The probe is placed on the cervix twice for three minutes each with a break of five minutes between each freeze [15]. This cryotherapy option allows for same-day diagnosis and treatment for women whose lesions do not extend to the endocervix or cover more than three quarters of the cervix [15]. LEEP must be available as back up for women with more significant lesions.

There has been limited clinical or economic research comparing LEEP and cryotherapy for treatment of cervical dysplasia among HIV-positive women. As policy makers in South Africa and other countries consider treatment options for HIV-positive women, both clinical and cost data may influence decision-making. Smith et al (2017) conducted one of the first randomized controlled trials comparing LEEP to cryotherapy for treatment of CIN 2+ among HIV-positive women in South Africa, most of whom were taking antiretroviral treatment [17]. In this analysis, we estimate the costs and evaluate the cost-effectiveness of these two treatment options as offered to the same cohort of HIV-positive women.

## Materials and methods

### Effectiveness

The effectiveness measures for this evaluation are derived from the randomized clinical trial completed by Smith et al. (2017) (clinicaltrials.gov NCT01723956) [17]. The trial was conducted from January 2010 to August 2014 at a large, outpatient HIV clinic located within a public tertiary hospital in Johannesburg. HIV-positive women aged 18–65 with a diagnosis of CIN2+ on colposcopic biopsy and lesions deemed eligible for treatment with cryotherapy were randomly assigned to either cryotherapy or LEEP at baseline. (Women not eligible for cryotherapy had lesions covering >75% of the cervix or lesions extending into the cervical canal resulting in borders not being visible.) At six months, both a conventional Pap smear and colposcopic biopsy were performed (i.e. at the same time). If CIN2+ was found on colposcopic biopsy at the six-month visit, the protocol specified that LEEP should be performed regardless of the initial treatment assignment. However, some women did not receive LEEP retreatment because they did not return for the treatment or were pregnant. Pap smear and colposcopic biopsy were both performed again at 12 months. Women with CIN2+ identified at 12 months were referred to a gynecologist for review for retreatment.

In the clinical trial, endpoints were presented as cumulative risk estimates at six months and at 12 months and included cytological and histological results. Women were censored from the analysis if they became pregnant, dropped out of the study, died, or if they had no result available at both the six and the 12-month visit [17].

Effectiveness for this cost-effectiveness analysis was defined *a priori* as “cure of CIN2+,” or the absence of disease categorized as CIN2 or greater, at 12 months after the initial treatment with cryotherapy or LEEP. We derived effectiveness outcomes in two ways. First, we assumed an intention-to-treat approach where all cases censored prior to their 12-month assessment due to the reasons noted above were considered to be treatment failures. We calculated simple proportions and 95% confidence intervals (CIs) for CIN2+ outcomes at six or 12 months using Stata’s Clopper-Pearson (i.e. exact) option (v14, StataCorp LP). Second, we used the as-treated cumulative effectiveness outcomes (i.e. based on all results obtained at six or 12 months) and CIs as reported for the clinical trial [17].

All women provided written consent to participate in the clinical trial. Ethics approval for the trial and this economic evaluation was obtained from the Human Ethics Research Committee at the University of the Witwatersrand.

### Costing

We conducted the cost evaluation from a health service perspective. We used micro-costing to collect cost data for the LEEP and cryotherapy procedures between June 2013 and April 2014 at the same health facility that implemented the clinical trial. We included personnel, consumables, equipment and laboratory costs. Infrastructural and overhead costs (e.g. space, electricity, security, etc.) were not included in the analysis as both procedures were provided in the same physical space at the clinic. Training on cryotherapy or LEEP and patients’ costs were also not included.

We collected information on the resources required for the procedures through discussions with facility staff. Two doctors and one professional nurse, who performed the treatment procedures, provided input regarding average times per procedure and information regarding supply and equipment usage. A third, senior doctor at the facility with oversight responsibility for the treatment procedures also provided input as needed.

Personnel costs and unit costs for consumables and equipment were obtained from facility expenditure records or other publicly available sources [18,19]. Laboratory costs represent service charges from the National Health Laboratory Service (NHLS), which provides laboratory services for the public sector in South Africa [20]. Equipment costs were annualized following generally accepted methods to obtain the equivalent annual cost using a discount rate of 3% and depreciation periods recommended for various categories of equipment by the South Africa Revenue Service [21,22]. We collected all costs in South African Rand (R) and inflated the costs to 2015 prices (where necessary) using the South African Consumer Price Index [23]. Costs are reported here in 2015 US dollars (US$) using an average exchange rate for 2015 of 12.77 Rands per dollar [24].

Cytology (conventional Pap smear) and colposcopic biopsies were also performed in the study. We have included the costs of these activities as these would be standard of care for follow-up after treatment of pre-cancerous lesions, and the colposcopic biopsy was required to determine the “cure” outcome required for this analysis at six and 12 months. The average costs per Pap smear and colposcopic biopsy as performed in the study clinic were published by this study team in 2015 [25]. The results were presented in 2013 US dollars and were inflated to 2015 US dollars for this analysis.

Finally, if CIN2+ was found at the six-month visit–for women assigned to cryotherapy or LEEP at baseline–and a LEEP was performed at six months, we included that LEEP cost in the 12-month total cost calculations. We excluded the costs of any care provided after the primary 12-month outcome was obtained. For example, for women found to have persistent CIN2+ at 12 months, we excluded the costs of the gynecologist review and retreatment if performed.

### Analysis

We entered resource usage and cost inputs into an Excel (Microsoft Corporation, 2010) model designed for this study. The model contained: 1) source data and unit costs, 2) resource usage data collected through the micro-costing activities, and 3) worksheets showing analytical work and outcomes. A full listing of the model contents is available in S1 Workbook snapshot.

**Fig 1:**
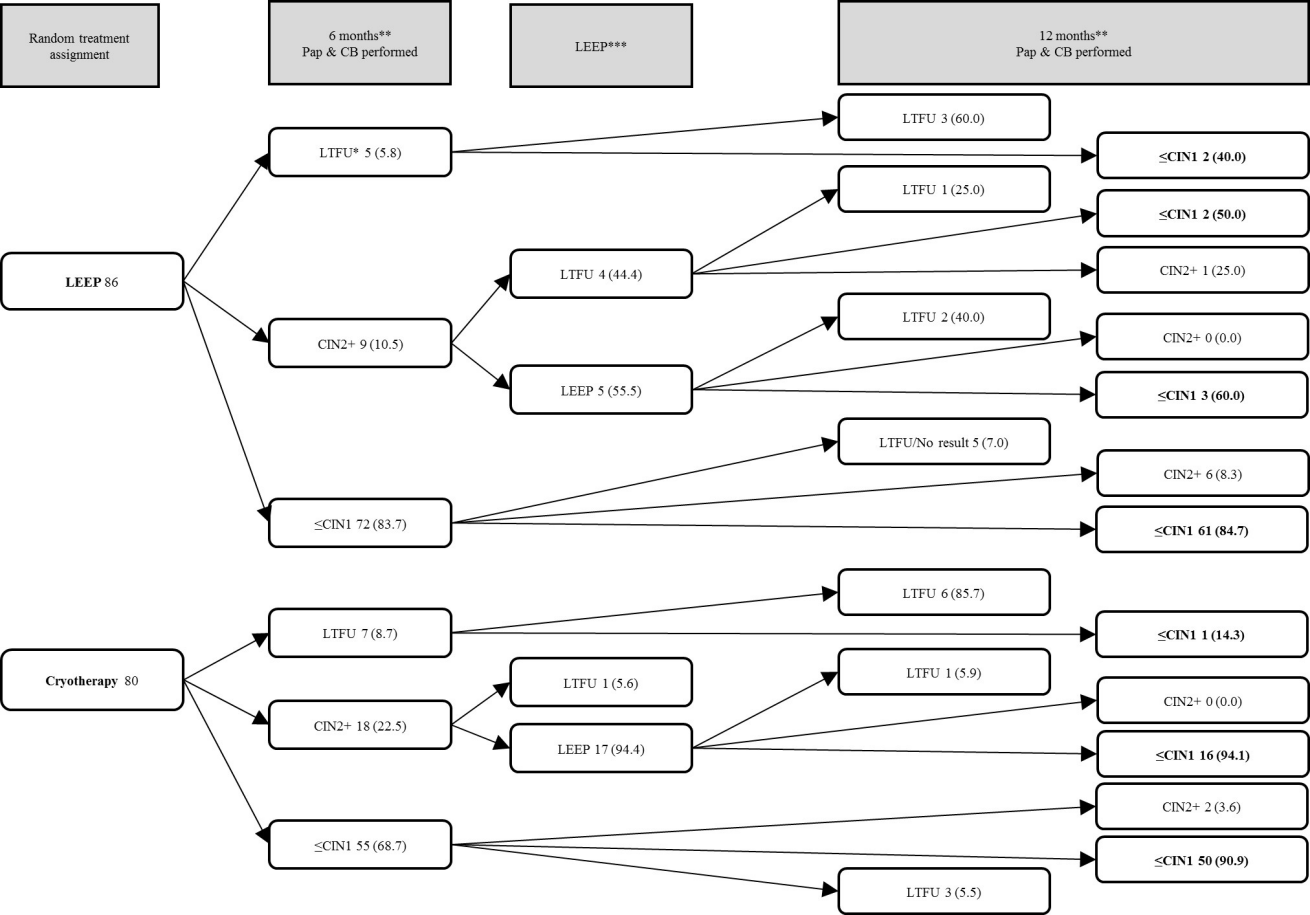
Treatment assignment, 6- and 12-month follow-up rates and outcomes for HIV-positive women receiving LEEP or cryotherapy for CIN2+ in a clinical trial in Johannesburg, South Africa (n (%)) [17]. CB = colposcopic biopsy; Pap = conventional cytology; CIN = Cervical intraepithelial neoplasia; CIN2+ = CIN level 2 or greater; ≤CIN1 = No disease or CIN1; LTFU = lost to follow-up. * Women could be LTFU at 6 months, indicating a missed visit, for their LEEP appointment after the 6-month visit, or at 12 months, meaning that no outcome was obtained at 12 months. In the clinical trial [17] and the as-treated analysis, women were censored if they were pregnant, formally left the study, or died. In the intention-to-treat analysis, women who were LTFU at 12 months were classified as treatment failures. **Colposcopic biopsies and conventional cytology were performed at six and 12 months to evaluate the presence of disease. ***At 6 months, LEEP was performed for women with CIN2+ (unless the woman did not return for treatment or was pregnant) regardless of the initial treatment assignment.

Within the model, we performed decision tree analysis to calculate the total cost per treatment group, the total average cost per case treated, and the total average cost per case of CIN2+ “cured” in each treatment group–LEEP or cryotherapy. Fig 1 illustrates the possible patient pathways as reported for the clinical trial [17].

During the clinical trial, the number of women treated with cryotherapy or LEEP on a monthly basis was lower than the total number of women treated at the study facility. To explore the impact of service volume on the total and average cost outcomes, we created two caseload scenarios. The Study Statistics scenario uses the average number of procedures actually done per day per medical officer, or doctor, during the clinical trial. The Functional Limit scenario represents the maximum number of procedures estimated to be possible per doctor during an eight-hour work day given the time required per procedure, which includes time spent with each patient as well as daily, weekly and monthly tasks required for the service. To address uncertainty regarding the costs of equipment, supplies and staff time per procedure, we have varied these inputs by ±25% and presented the results as a range around the base case cost estimates for each treatment group under both service volume scenarios.

We then used the cost outcomes from the Functional Limit scenario and treatment outcomes reported in the clinical trial to calculate an Incremental Cost Effectiveness Ratio (ICER) (i.e. the difference in intervention costs divided by the difference in intervention outcomes). As the treatment groups were not equivalent in size, the proportion of cases cured for each treatment group was applied to a hypothetical population of 100 women (i.e. proportion cured x 100) to ensure that the difference in treatment group sizes did not bias the ICER calculation. Likewise, total costs for each intervention group were determined for the hypothetical population of 100 patients (i.e. average cost per outcome x 100 patients).

Finally, to address the possible effects of uncertainty in both the cost outcomes and the clinical outcomes from the clinical trial on the ICER, we performed deterministic univariate sensitivity analysis using the ranges calculated for the costs and the confidence intervals for the CIN2+ outcomes at 12 months. The ICER was also re-calculated using the as-treated results from the clinical trial.

We consulted the Consolidated Health Economics Evaluation Reporting checklist, a standardized, internationally recognized tool, in preparing this manuscript [26].

## Results

In total, 166 women with CIN2+ disease were enrolled in the clinical trial and randomized to LEEP (n = 86) or cryotherapy (n = 80) [17]. Fig 1 provides the numbers of women who had each treatment, were lost to follow-up, and for whom a final outcome was assessed. Table 1 presents the proportions of women who returned for the six and 12-month assessments and the proportions with CIN2+ diagnosed at each time point. Loss to follow-up affected the two study arms differentially. At 12 months, cumulatively 12.8% (11/86) of participants in the LEEP group were lost to follow-up as compared to 13.8% (11/80) in the cryotherapy group.

**Table 1:** Treatment outcomes at 6- and 12-months, intention-to-treat and as-treated [17] analyses (% (n/N)).

	Treatment group			
	LEEP		Cryotherapy	
	% (n/N)	95% CI*	% (n/N)	95% CI*
**Intention-to-treat analysis**				
Treated for CIN2+ at baseline	100.0 (86/86)	—	100.0 (80/80)	—
*6 months*				
Returned for 6-month visit	94.2 (81/86)	—	91.2 (73/80)	—
If returned, CIN2+ diagnosed	11.1 (9/81)	(5.2–20.1)	24.7 (18/73)	(15.3–36.1)
CIN2+ retreated with LEEP	55.6 (5/9)	—	94.4 (17/18)	—
*12 months*				
Returned for 12-month visit	87.2 (75/86)	—	86.3 (69/80)	—
CIN2+ diagnosed at 12 months and …:				
≤CIN1 at 6 months	8.3 (6/72)	(3.1–17.3)	3.6 (2/55)	(0.4–12.5)
CIN2+ at 6 months, LEEP at 6 months	0.0 (0/5)	(0.0–52.2)**	0.0 (0/17)**	(0.0–19.5)**
CIN2+ at 6 months, no LEEP at 6 months	25.0 (1/4)	(0.6–80.6)	0.0 (0/1)**	(0.0–97.5)**
“Cured” at 12 months***	79.1 (68/86)	(69.0–87.1)	83.8 (67/80)	(73.8–91.1)
**As-treated analysis** [17]				
Cumulative CIN2+ incidence as reported in clinical trial****	18.1 (15/83)	(10.5–28.1)	27.0 (20/74)	(17.4–38.6)
Absence of disease at 6 or 12 months[17]*****	81.9		72.9	

“Cured” = absence of CIN2+

* Two-sided 95% confidence interval (CI) unless otherwise noted.

**One-sided 97.5% confidence interval.

***Assumes all cases that were lost to follow-up at 12 months have disease. The denominator includes participants who were censored in the clinical trial.

**** Calculated using a denominator that represents only those women who were not censored at 12 months.

***** Inverse of the cumulative CIN2+ incidence

Based on the unadjusted intention-to-treat analysis conducted for this economic evaluation, there was no significant difference in efficacy for the two interventions at 12 months of study follow-up. However, the “cure rate” (or proportion of women free from CIN2+) in the cryotherapy arm at 12 months was higher than the rate in the LEEP arm. Of women with CIN2+ at baseline and randomized to cryotherapy, 83.8% (67/80) (95% CI 73.8–91.1) were found to be free of CIN2+ disease at 12 months. In comparison, 79.1% (68/86) (95% CI 69.0–87.1) of women assigned to LEEP were found to be free from disease at 12 months.

This contrasts with the results presented for the clinical trial, where cumulative incidence (i.e. cases of CIN2+ cumulatively diagnosed at up to six and up to 12 months) was compared across the two trial arms, and women were censored from the final, 12-month analysis if they were pregnant, exited the study, died prior to 12 months or missed visits at both six and 12 months. In the as-treated analysis conducted for the clinical trial, cumulative incidence of CIN2+ at 12 months was higher among women who had cryotherapy at baseline (27.0% (20/74) (95% CI 17.4–38.6)) than among women who had LEEP (18.1% (15/83) (95% CI 10.5–28.1)) [17]. This difference was again not statistically significant.

### Resource utilization

The resources required to provide treatment are summarized in Table 2. The LEEP procedure required both a medical officer, or doctor, and a professional nurse to complete the procedure, while cryotherapy was performed by a “Primary Health Care” nurse.

**Table 2:** Resources utilized to provide LEEP and cryotherapy services within an HIV care and treatment facility in Johannesburg, South Africa*.

Resource	Details
Personnel^**^	
Staff nurse	Retrieved the day’s supplies, set up the rooms, and provided support as needed
Professional nurse	Assisted with LEEP
PHC nurse	Performed cryotherapy
Medical officer	Performed LEEP procedures
Counselor	Assisted in calling back women and scheduling return visits if needed
*Supplies and equipment*	
Supplies	Gloves, masks, linen savers, cotton swabs, paper towels, hand washing/sanitizing supplies, pens/pencils, forms, files, sanitary towels, acetic acid, paper towels, etc. Nitrous oxide gas for cryotherapy.
Furnishings	Exam table with stirrups, theater bed, trolley, desks, etc.
Other equipment	Speculum (insulated and metal), cryotherapy gun, cryotherapy probe, monitor, LEEP machine, loops, etc.
*Laboratory*	
Histology	NHLS laboratory fees which included LEEP specimen vials, formalin, lab forms, materials for shipment to/from laboratory and processing and reporting of the results of the biopsy specimen

NHLS = National Health Laboratory Service; PHC nurse = Primary health care nurse; Medical officer = doctor

*Resources required for screening (Pap smear and colposcopic biopsy) have been published previously [25].

**Training for the nurses is as follows: Staff nurse, 2 years; Professional nurse, 4 years; PHC nurse, 6 years.

### Service volume and treatment costs

Table 3 provides information on expected service volume under the two caseload scenarios. Under the Study Statistics scenario, far fewer procedures were performed per day for both cryotherapy and LEEP. This is due to a slow enrollment rate and long follow-up period for patients in the study. As explained above, the Functional Limit scenario represents the number of procedures possible per a hypothetical eight-hour work day. Under the Functional Limit scenario, 18.6 LEEP and 11 cryotherapy procedures could be performed per day. While these numbers might not be possible due to interruptions expected in a typical work day, they can be considered as a hypothetical maximum upper bound. Likewise, the study statistics represent very low service volume as a result of the study being performed only with eligible study participants booked for services on specific days. Actual service volume at the facility was higher than the study statistics scenario, which represents a lower bound.

**Table 3:** Caseload scenarios: Total procedures per day per study nurse or doctor.

Scenario	LEEP	Cryotherapy
Study statistics*	1.7	1
Functional limit**	18.6	11

*Average number of procedures done in the clinic during the study timeframe

**Calculated using the reported time required per procedure plus time required for daily, weekly and monthly activities directly required by each service. It assumes 8 hours of productive time per work day

Table 4 contains the average cost per procedure for both treatment methods under the two caseload scenarios. The costs were higher for both procedures in the Study Statistics scenario than in the Functional Limit scenario due to the low service volumes in the Study Statistics scenario. Comparing the two treatment procedures, cryotherapy was less costly than LEEP in both scenarios. The total average cost per women for cryotherapy in the Study Statistics scenario was US$ 15.59 (Uncertainty interval (UI) 11.69–19.49); for LEEP it was at US$ 73.78 (UI 67.24–80.32). In contrast, in the Functional Limit scenario, the total average cost of LEEP was US$ 54.11 (UI 52.49–55.73). This was more costly than the average cost for cryotherapy at US$ 3.46 (UI 2.60–4.33) in the same scenario. For both scenarios, the difference in intervention costs is explained by the lack of laboratory costs for cryotherapy.

**Table 4:** Average estimated procedure costs, with cost breakdown, for each service volume scenario (USD 2015), intention-to-treat analysis.

	Functional Limit			Study Statistics		
	Cost	(Uncertainty interval)*	% of total	Cost	(Uncertainty interval)*	% of total
**LEEP**						
Personnel	1.94	(1.46–2.43)	3.6%	2.23	(1.67–2.79)	3.0%
Supplies	2.72	(2.04–3.40)	5.0%	3.89	(2.91–4.86)	5.3%
Equipment	1.83	(1.37–2.29)	3.4%	20.04	(15.03–25.05)	27.2%
Laboratory	47.62	(47.62–47.62)	88.0%	47.62	(47.62–47.62)	64.5%
**Total**	**54.11**	**(52.49–55.73)**	**100.0%**	**73.78**	**(67.24–80.32)**	**100.0%**
**Cryotherapy**						
Personnel	1.45	(1.09–1.81)	41.9%	2.46	(1.85–3.08)	15.8%
Supplies	0.99	(0.74–1.23)	28.4%	1.85	(1.39–2.32)	11.9%
Equipment	1.03	(0.77–1.28)	29.6%	11.27	(8.45–14.09)	72.3%
Laboratory	0.00	—	0.0%	0.00	—	0.0%
**Total**	**3.46**	**(2.60–4.33)**	**100.0%**	**15.59**	**(11.69–19.49)**	**100.0%**

*Cost ranges represent uncertainty analysis (i.e. ±25% for staff time and supply and equipment costs). Laboratory costs per LEEP procedure are not varied because they are charges by the National Health Laboratory Service to the state. They are negotiated, published rates (and are thus certain).

### Total costs and cost-effectiveness

Assuming average procedure costs as calculated for the Functional Limit scenario, Table 5 provides a summary of the total costs for treatment and diagnostics in each study arm as well as the cost per “cured” patient at 12 months. The total cost for treatment and diagnostics in the LEEP group at 12 months was US$ 13,980.32, and for the cryotherapy group it was US$ 9,440.37. On average, cryotherapy was less costly per patient randomized at US$ 118.00 (UI 113.91–122.10), and per case “cured” at US$ 140.90 (UI 136.01–145.79). LEEP cost US$ 162.56 (UI 157.90–167.22) per patient randomized and US$ 205.59 (UI 199.70–211.49) per case cured.

**Table 5:** Total and average costs of treating analytical cohort for CIN 2+ (US$ 2015) (Functional Limit scenario), intention-to-treat analysis.

	LEEP (N = 86)		Cryotherapy (N = 80)	
	Cost	Uncertainty interval	Cost	Uncertainty interval
**Total costs**				
Baseline–initial treatment	4,653.55	4,514.08–4,793.02	277.01	207.76–346.26
*6 months*				
Diagnostics*	4,702.26	4,570.67–4,833.85	4,237.40	4,119.25–4,356.43
LEEP re-treatment	270.56	262.45–278.66	919.89	892.32–947.46
*12 months*				
Diagnostics*	4,353.95	4,232.11–4,475.79	4,005.22	3,893.54–4,117.72
Total costs**	13,980.32	13,579.31–14,381.32	9,440.37	9,112.86–9,767.88
**Average cost**				
Per patient randomized	162.56	157.90–167.22	118.00	113.91–122.10
Per case “cured” at 12 months***	205.59	199.70–211.49	140.90	136.01–145.79

“Cured” = absence of CIN2+

*Diagnostic costs include Pap smears and colposcopy biopsies.

** Total = initial treatment + diagnostics at 6 and 12 months + LEEP at 6 months if needed

*** Under an intention-to-treat analysis where all lost to follow-up cases are considered to have had disease.

Table 6 presents the results of the cost-effectiveness analysis, including the ICER. LEEP was strongly dominated by cryotherapy as cryotherapy was both more effective and less costly.

**Table 6:** Cost-effectiveness analysis comparing cryotherapy versus LEEP for treatment of CIN2+ among HIV-positive women (US$ 2015), intention-to-treat analysis.

Strategy	Total cost*	Incremental cost		Effectiveness*	Incremental effectiveness	ICER
Cryotherapy	11,800.47	—		84		
LEEP	16,256.18	4,455.72		79	-5	Dominated

*Based on a hypothetical population of 100 individuals per arm.

Univariate deterministic sensitivity analysis, where we varied the costs of the diagnostic procedures as presented in Table 4 and the confidence intervals for incidence of CIN2+ as presented in Table 1, showed that under all conditions but one, cryotherapy continued to strongly dominate LEEP services. When the incidence of CIN2+ in the cryotherapy treatment arm for patients who were negative at six months was raised to the limit of its 95% confidence interval, LEEP was no longer dominated by cryotherapy. However, cryotherapy remained more cost effective.

Tables 7 and 8 show sensitivity analysis results using the as-treated, or cumulative incidence, outcomes as reported for the clinical trial [17]. As in the intention-to-treat analysis, the average cost per case “cured” at 12 months remained lower for cryotherapy than for LEEP. However, LEEP, again, was no longer dominated in cost-effectiveness analysis. Cryotherapy remained more cost effective, however, with an ICER per disease free case of US$ 497.85.

**Table 7:** Average cost per disease-free case (US$ 2015), as-treated analysis.

	Cost	Range
Cryotherapy (N = 80)	161.87	156.26–167.49
LEEP (N = 86)	198.49	192.79–204.18

**Table 8:** Cost-effectiveness (US$ 2015), as-treated analysis.

	Total cost*	Incremental cost	Effectiveness*	Incremental effectiveness	ICER
Cryotherapy	11,800.47	—	73	—	—
LEEP	16,256.18	4,455.72	82	8.95	497.85

*Based on a hypothetical population of 100 individuals per arm.

## Discussion

The clinical trial conducted by Smith et al (2017) compared LEEP versus cryotherapy for the treatment of CIN2+ disease among HIV-positive women, most of whom were taking antiretroviral treatment [17]. The trial results, based on an as-treated analysis, showed LEEP to be more efficacious than cryotherapy; however, the difference in outcomes was not statistically significant at 12 months [17].

In this economic evaluation, we evaluated and compared the cost-effectiveness of the same two treatment approaches, using an intention-to-treat analysis, where women who were lost to follow-up were considered to be treatment failures at 12 months. In this analysis, cryotherapy (plus LEEP if needed at six months) appeared to be a more cost-effective approach than LEEP (plus an additional LEEP if needed) under all conditions. The cryotherapy-plus-LEEP treatment option resulted in an average cost of US$ 118.00 per case “cured” at 12 months.

Interpreting these cost-effectiveness results, and their seeming contradiction to the clinical study’s outcomes, for policy relevance requires careful unpacking. There is little published guidance on the appropriate method for economic evaluation in cases such as these where no significant difference in treatment outcomes exists. It has however been suggested that unless trials are designed specifically to show that treatments are equivalent, a cost-minimization approach is best avoided, and cost-effectiveness should rather be estimated [27].

Pap smear followed by colposcopic biopsy and LEEP, if needed, is currently the standard of care in South Africa [11]. However, in actuality, access and utilization of services are poor. National statistics show that in 2014, just 54.5% of the target population for screening had a Pap smear [28], and the last Demographic and Health Survey for the country indicated that for women in the poorest wealth quintile, just 12.8% had a pelvic examination and Pap smear in the past three years [29]. Statistics regarding access to colposcopic biopsy and LEEP are not available; however, access is known to be very poor with long waiting times to obtain an appointment, and some provinces offering these in just one or no locations.

In their clinical trial, Smith et al (2017) reported lower than expected efficacy of both cryotherapy and LEEP when evaluated among HIV-positive women in South Africa compared to HIV uninfected women [17]. The authors suggested that HIV-positivity and high HPV prevalence in the study cohort may have contributed to the poor outcomes, and they called for future research examining the efficacy of other treatment options in HIV-positive women. We concur.

There is limited literature on the costs and cost-effectiveness of cryotherapy and LEEP, particularly in Africa [30]. A study on the costs of visual inspection with acetic acid followed by cryotherapy in Ghana estimated the cost of cryotherapy treatment alone varied between $47.26 and $84.48 (US 2009) per woman at different facilities, which is substantially higher than our finding of $3.46, for the Functional Limit scenario [31]. Unlike our study, the Ghanaian study included overhead costs, but this likely does not account for all of the large difference. A study in rural China in 2008 estimated the costs of LEEP to be $61.38 per patient treated [32], which is somewhat higher than our estimated cost under the Functional Limit scenario.

This economic evaluation has limitations. The costs represent service provision norms in South Africa, including nurse provision of cryotherapy. Training costs and overheads were excluded. As the evaluation takes a health service perspective, patients’ costs were also excluded. Cryotherapy (without LEEP follow-up) can be offered on the same day as VIA for screening and treatment in primary care facilities, and thus may be more convenient (and less costly) for patients. However, VIA is not offered in South Africa’s public sector. The evaluation also stops at an intermediate outcome–“case cured at 12 months”–and does not consider the potential longer term costs, morbidity and mortality associated with progression to cancer among women who were not successfully treated.

Despite these limitations, we feel that this evaluation may offer insight to policy makers in HIV-endemic areas who are faced with questions about the best approach for treating cervical dysplasia. Globally, nearly 65% of all cancer-related deaths happen in less developed regions [33]. In contrast, just 5% of global spending on cancer occurs in low- and middle-income countries [34], likely as a result of limited funding. Any environment with high HIV prevalence, and particularly those with concomitantly high HPV prevalence, requires a national cervical cancer screening and treatment program that allows for frequent screening and timely access to cost effective treatment and re-treatment if needed.

Revisions to South Africa’s national policy for screening and treatment of cervical cancer were finalized in 2017. LEEP services remain the treatment option of choice in the country despite their higher cost [12]. Based on their clinical trial, Smith et al (2017) noted that neither LEEP nor cryotherapy was an optimal treatment option for HIV-positive women, and that further research was needed. This economic analysis suggests that initiating treatment with cryotherapy for CIN2+ women and following up with LEEP at six months, if needed, may be a more cost-effective treatment option than LEEP alone for HIV-positive women. However, this is only an option for women eligible for cryotherapy. In the study by Smith et al (2017), nearly 60% of women with CIN 2+ who were recruited for the study were found to be ineligible for cryotherapy. Further research is certainly needed to identify more effective treatment options for CIN2+ among HIV-positive women, and in the meantime, South African policy makers should ensure access to existing treatment in a timely manner and provide careful and conscientious monitoring of treatment outcomes over time.

## Supporting information

S1 Workbook Table of contents—VICAR II CEAs. This snapshot of the model table of contents illustrates the data contained and how it is manipulated.
(PDF)
