# Correction: Costs and cost-effectiveness of LEEP versus cryotherapy for treating cervical dysplasia among HIV-positive women in Johannesburg, South Africa

**DOI:** 10.1371/journal.pone.0212391

**Published:** 2019-02-12

**Authors:** Naomi Lince-Deroche, Craig van Rensburg, Jacqueline Roseleur, Busola Sanusi, Jane Phiri, Pam Michelow, Jennifer S. Smith, Cindy Firnhaber

The third author’s name is spelled incorrectly. The correct name is: Jacqueline Roseleur.
