# Supplementary material for: Costs and cost-effectiveness of LEEP versus cryotherapy for treating cervical dysplasia among HIV-positive women in Johannesburg, South Africa
Source: PLoS One. 2018 Oct 11;13(10):e0203921. doi: 10.1371/journal.pone.0203921 (PMC6181291; doi:10.1371/journal.pone.0203921)
Supplement: S1 Workbook — This snapshot of the model table of contents illustrates the data contained and how it is manipulated. (PDF) [file pone.0203921.s001.pdf]

Table of Contents - VICAR II CEAs

NB: Contents listing is hyperlinked to workbook tabs.

**Functional and analytical worksheets**	***Description***
Cost summary	*Summary of costs for all procedures, with breakdown*
Decision tree for cost-effectiveness calculations	*Clinical and service parameters X average costs per activity, including unscheduled visits*
Average cost per activity calculations	*Resource usage x unit costs per resource for abortion procedure activities (does not include unscheduled visits)*
Analysis parameters	*Listing of parameters (analysis year, discount rate, etc) which can be varied if desired*
Sensitivity Analysis	*Ranges for adjustments for sensitivity analysis*
**Research usage and clinical outcomes data**	***Source***
Clinical service parameters and outcomes	*Study database, provider interviews*
Service volume for scenarios	*Study enrollment records, clinic records*
Staff time - Pap (conventional)	*Provider interviews*
Staff time - Colposcopic biopsy	*Provider interviews*
Staff time - LEEP	*Provider interviews*
Staff time - Cryotherapy	*Provider interviews*
Consumable costs	*Provider interviews, observation*
Equipment costs	*Provider interviews, observation*
Labs/diagnostics usage and costs	*Provider interviews, observation, National Health Laboratory Service*
**Appendices: Unit cost and other source information**	***Source***
Depreciation, exchange rates, etc.	*IMF, World Bank, Statistics South Africa, etc*
Inflation	*IMF, World Bank, Statistics South Africa, etc*
Personnel costs (per type and per minute)	*Calculated using Department of Public Service Administration (DPSA) and general accounting practice guidelines for South Africa*
Supply prices	*National tenders and other sources*
Equipment prices	*National tenders and other sources*
